# Prognostic factors associated with recurrence in patients undergoing surgery for hepatic cystic echinococcosis: A systematic review protocol

**DOI:** 10.1371/journal.pone.0329235

**Published:** 2025-08-06

**Authors:** Josue Rivadeneira, Luis Fuenmayor-González, Anis Hasnaoui, Diomaris Ortiz, Gabriela Zambrano, Claudio Rojas-Pincheira, Carlos Manterola

**Affiliations:** 1 Universidad de La Frontera, Doctorado en Ciencias Médicas, Temuco, Chile; 2 Zero Biomedical Research, Quito, Ecuador; 3 Unidad de Revisiones Sistemáticas y Metaanalisis-URMA, Facultad de Ciencias Médicas, Universidad Central del Ecuador, Quito, Ecuador; 4 Faculty of Medicine of Tunis, Tunis El Manar University, Tunis, Tunisia,; 5 Hospital de Especialidades Eugenio Espejo, Quito 170403, Ecuador; National Research Centre, EGYPT

## Abstract

**Background:**

Cystic echinococcosis (CE) is a zoonotic disease caused by *Echinococcus granulosus*, with hepatic localization being the most frequent presentation in humans (70–80%). Surgical intervention remains the most common therapeutic option, despite an associated recurrence rate of 8% (95% CI: 6%–10%). CE recurrence increases postoperative morbidity, hospital stay, and healthcare costs. Several prognostic factors (PFs), such as cyst size, presence of multiple cysts, and personal or family history of CE, have been linked to recurrence. However, associations with cyst location, type, and biliary complications remain uncertain due to conflicting evidence. This systematic review (SR) aims to identify PFs associated with recurrence in patients undergoing surgery for hepatic CE.

**Methods:**

We will conduct a systematic review of primary longitudinal observational, and experimental studies evaluating the association between clinical, parasitic, and surgical PFs and CE recurrence. We will search Medline, Scopus, Embase, Web of Science, BIREME-BVS, and SciELO using a sensitive search strategy. Two reviewers will independently screen studies, extract data, and assess risk of bias using the QUIPS tool. Methodological quality will be assessed using MInCir-Pr2, and data extraction will follow the CHARMS-PF checklist. A qualitative synthesis will be performed, and where appropriate, random-effects meta-analyses will be conducted using the inverse-variance and restricted maximum likelihood methods. Heterogeneity will be assessed using Chi², Tau², I², and 95% prediction intervals. Publication bias will be explored via funnel plots and statistical tests (Egger’s). Certainty of evidence will be assessed using GRADE.

**Discussion:**

The results of this SR will support early risk stratification and inform clinical decision-making in the management of hepatic CE, a neglected tropical disease.

**Registration:**

PROSPERO, CRD42024538005.

## Background

Cystic echinococcosis (CE) or hydatidosis is a zoonotic disease caused by the metacestode of the parasite *Echinococcus granulosus (E. granulosus)*. CE is considered one of the twenty neglected tropical diseases by the World Health Organization (WHO) [1] and one of the zoonoses with the highest burden on human and animal health [2]. Reported worldwide, except in Antarctica, it reaches incidence rates of up to 50 cases per 100,000 inhabitants in endemic regions of Africa, East Asia, the Middle East, and South America [3].

To 2021, CE was responsible for 0.58 disability-adjusted life years (DALYs) per 100,000 population, imposing a significant disease burden, especially in low-income countries [4]. The global underdiagnosis and underreporting of cases hinder accurate estimation of the disease’s prevalence and incidence and its associated economic losses. However, in endemic areas such as India or Chile, CE has been associated with high medical costs (USD 8.75 million and USD 3.13 million per year, respectively), mainly due to surgical procedures, hospital stays, and medical licenses, among other factors [5,6].

In humans, an accidental intermediate host, the liver is the most frequently affected organ (70–80%) [7]. Characterized by a silent, progressive, and chronic course, hepatic cystic echinococcosis (HCE) remains asymptomatic for long periods and is usually diagnosed incidentally through imaging techniques such as abdominal ultrasound (AUS), computed tomography (CT), and magnetic resonance imaging (MRI). These tests describe the structural characteristics of the cysts, allowing for classification, staging, and treatment planning according to the recommendations of the WHO Informal Working Group on Echinococcosis (WHO-IWGE) [8,9].

Regarding treatment, therapeutic options for HCE range from a “watch and wait” strategy to surgical removal of the cyst, depending on the stage, viability, and presence of evolutionary complications of the cyst [10]. Surgical intervention -which has a recurrence rate of 8% (95% confidence interval [CI]: 6%–10%) [11]- is the only curative treatment and the most widely used therapeutic approach. Each surgical technique carries a different risk of recurrence, depending on the type of access, extent of cyst resection, and management of the residual cavity (Table 1) [12–16].

**Table 1:** Risk of CE recurrence by surgical technique. Results from systematic reviews.

Surgical Intervention	Effect Measure	Effect Size (95% CI)	N° of Participants (N° Studies and design)	Author
**Type of access:** Laparoscopy vs. Open	OR	0.90 (0.16–4.90)	785 (3 Observational)	Sokouti, 2017 [13]
**Type of resection:** Radical vs. Conservative	OR	0.16 (0.11–0.23)	3.944 (19 Observational)	Pang, 2018 [14]
	OR	0.17 (0.08–0.38)	1.267 (5 Observational)	He, 2015 [15]
**Cavity management:** Omentoplasty vs. External drainage	OR	0.16 (0.01–3.31)	53 (1 RCT)	Dziri, 2022 [16]
	OR	0.28 (0.04–2.22)	444 (4 Observational)	
	OR	0.24 (0.04–1.30)	497 (1 RCT and 4 Observational)	

*OR: Odds ratio; 95% CI: 95% Confidence Interval; RCT: Randomized Controlled Trial.*

Several clinical features have been identified as predictive factors for CE recurrence: a family history of CE (odds ratio [OR]: 214; 95% CI: 26.4–1731.7), previous surgery for CE (OR: 2.2; 95% CI: 1.2–3.9), cyst size (OR: 4.44; 95% CI: 1.83–10.80), presence of multiple cystic lesions (OR: 3.8; 95% CI: 2.0–6.9), and cyst rupture indicated by anaphylaxis (OR: 15.3; 95% CI: 1.34–174.1) [17,18]. However, the potential associations between recurrence and cyst location within liver segments or depth, presence of extrahepatic cysts, and diagnosis of biliary complications remain inconclusive due to inconsistent findings requiring consensus for clinical application [18–20].

Compared to patients without recurrence, those with recurrent CE experience higher postoperative morbidity (biliary complications: 100% vs. 6.1%, p < 0.01) [17], longer hospital stays (16.4 vs. 9.5 days; p = 0.018) [21] and increased healthcare costs. Identifying preoperative and intraoperative prognostic factors (PFs) would enable early risk stratification in patients undergoing surgical treatment for HCE, facilitating individualized follow-up and therapy, and reducing disease-related costs. This underscores the need for a rigorous and structured systematic review to consolidate and critically appraise the available evidence.

Our systematic review will bring forth valuable insights by identifying prognostic factors associated with recurrence in patients undergoing surgery for HCE.

## Methods

**Protocol and registration:** This systematic review (SR) protocol was developed a priori following the Preferred Reporting Items for Systematic Review and Meta-Analysis Protocols (PRISMA-P) checklist (S1 Table) [22] and is registered in the PROSPERO database, on May 3, 2025, under the code CRD42024538005. The results will be reported following the Preferred Reporting Items for Systematic Reviews and Meta-Analyses 2020 (PRISMA) statement [23]. Table 2 describes the status and timeline of the study.

**Table 2:** Status and timeline of the systematic review.

Review stage	Started	Completed	Timeline
Pilot work	Yes	Yes	May 2025
Formal searching/study identification	Yes	No	June 2025
Screening search results against inclusion criteria	No	No	July 2025
Data extraction or receipt of IP	No	No	August 2025
Risk of bias/quality assessment	No	No	September 2025
Data synthesis	No	No	November 2025

**Eligibility Criteria:** Eligibility criteria are described using the PICOT acronym.

**Study design:** We will include primary observational longitudinal studies (prospective or retrospective) and experimental studies that assess the association between preoperative and surgical characteristics and the development of recurrence in patients who underwent surgery for HCE. No restrictions will be applied regarding geography, language, or publication date.

We will exclude case reports due to their high risk of selection bias, and letters to the editor that do not provide primary data. Cross-sectional studies will also be excluded as they are not suitable for assessing PFs. Systematic reviews on CE will not be included as primary sources but will be used for reference mining during manual searches.

**Population:** We will include primary studies that describe patients of any age, sex, and geographic location who underwent surgical treatment for HCE, diagnosed using one or more of the methods imaging (AUS, CT, or MRI); immunodiagnosis (detection of *E. granulosus* antibodies by ELISA, Western Blot, or hemagglutination); pathological confirmation following surgery; microscopic identification of protoscolex in hydatid fluid or medical record diagnosis.

We will exclude studies involving co-infection with other parasites or with different types of *Echinococcus*. Similarly, cases of isolated primary extra-abdominal CE (e.g., pulmonary, cerebral) are at increased risk of selection bias. However, studies that report separate results for the relevant subset will be considered eligible.

**Prognostic Index:** The initial search will be open-ended, including all variables reported as potential PFs. We will include studies describing PFs associated with CE recurrence. These variables will be classified as preoperative or intraoperative according to the time of measurement, though overlap may occur and will be defined based on the original studies.

Preoperative factors, assessed at study entry, diagnosis, or before surgery, will include the clinical characteristics obtained during routine history taking, including sex, presence/absence of comorbidities (e.g., diabetes, hypertension, dyslipidemia, asthma), family and personal history of CE. Also, the recorded signs and symptoms such as abdominal pain, palpable mass, jaundice, anaphylaxis, and others.

Likewise, parasite-related characteristics obtained via imaging, for example, cyst location, cyst size (>9 cm vs. ≤ 9 cm), cyst stage (WHO-IWGE classification), and HCE with any evolutionary complication, and others. Or laboratory findings from cyst puncture, such as the presence of bilirubin, alkaline phosphatase, and others.

Intraoperative factors, assessed during or after surgery, will include length of hospital stay (>3 days vs. ≤ 3 days), ICU admission, presence/absence of postoperative complications, biliary fistula, biliary communication, infection, cyst rupture, and others.

**Outcome:** The outcome of interest is the presence or absence of CE recurrence. We will use the definitions provided by each included study to reflect the variability in the literature [24].

CE recurrence will be confirmed through imaging (AUS, CT, or MRI), intraoperative diagnosis, postoperative pathological confirmation, microscopic identification of protoscolex in hydatid fluid, or any method proposed in the primary study. Recurrence will be assessed at any point during follow-up after surgery.

**Information sources:** Databases to be searched include Medline, Scopus, Embase, and Web of Science (WoS), as well as the virtual libraries BIREME-BVS and SciELO. We will perform manual reference searches of included studies and gray literature searches. Primary studies included in related SRs will also be reviewed.

**Search strategy:** We will perform sensitive searches adapted to each information source using MeSH, DeCS, and Emtree terms, as well as free-text keywords, combined using the Boolean operators OR and AND (Table 3). The search strategy was reviewed independently by two authors using the Peer Review of Electronic Search Strategies (PRESS) checklist [25].

**Table 3:** Block-based search strategy for the PubMed search engine (Medline).

Acronym	Blocks	Medline Search Terms
Population	1)	(“echinococcosis, hepatic”[MeSH Terms] OR “echinococcosis hepatic”[Title/Abstract] OR “echinococcus liver cyst”[Title/Abstract] OR “hepatic echinococcal cyst”[Title/Abstract] OR “Hepatic Echinococcosis”[Title/Abstract] OR “hydatidosis hepatic”[Title/Abstract] OR “Hepatic Hydatidosis”[Title/Abstract] OR “cysts hepatic hydatid”[Title/Abstract] OR “hepatic hydatid cyst*”[Title/Abstract] OR “Cystic Echinococcosis”[Title/Abstract] OR “echinococcus granulosus infection*”[Title/Abstract] OR “infection echinococcus granulosus”[Title/Abstract] OR “Liver Hydatid Cyst”[Title/Abstract] OR “Liver Hydatidosis”[Title/Abstract] OR “Hydatid Liver Disease”[Title/Abstract] OR “Hydatid Liver Cyst”[Title/Abstract] OR “echinococcal liver cyst”[Title/Abstract] OR “hepatic echinococcus cyst*”[Title/Abstract] OR “liver echinococcosis”[Title/Abstract] OR “liver hydatid disease”[Title/Abstract])
Index prognostic	2)	“Prognosis”[MeSH Terms] OR “Prognosis”[All Fields] OR “prognoses”[All Fields] OR “prognostic factor*”[All Fields] OR “factor prognostic”[All Fields]
Comparation	Not aplicable	
Outcome	3)	(“Recurrence”[MeSH Terms] OR “recurrence*”[Title/Abstract] OR “relapsing disease”[Title/Abstract] OR “recrudescence*”[Title/Abstract] OR “relapse*”[Title/Abstract] OR “recurrent*”[Title/Abstract] OR “Secondary echinococcosis”[Title/Abstract]))
Search string		(1) AND (3)
		(1)AND (2) AND (3)
Limits used	4)	NOT (“case reports”[Publication Type] OR “case report”[All Fields])) AND (humans[Filter])
**Final search string**	(1) AND (3) AND (4)	(((“echinococcosis, hepatic”[MeSH Terms] OR “echinococcosis hepatic”[Title/Abstract] OR “echinococcus liver cyst”[Title/Abstract] OR “hepatic echinococcal cyst”[Title/Abstract] OR “Hepatic Echinococcosis”[Title/Abstract] OR “hydatidosis hepatic”[Title/Abstract] OR “Hepatic Hydatidosis”[Title/Abstract] OR “cysts hepatic hydatid”[Title/Abstract] OR “hepatic hydatid cyst*”[Title/Abstract] OR “Cystic Echinococcosis”[Title/Abstract] OR “echinococcus granulosus infection*”[Title/Abstract] OR “infection echinococcus granulosus”[Title/Abstract] OR “Liver Hydatid Cyst”[Title/Abstract] OR “Liver Hydatidosis”[Title/Abstract] OR “Hydatid Liver Disease”[Title/Abstract] OR “Hydatid Liver Cyst”[Title/Abstract] OR “echinococcal liver cyst”[Title/Abstract] OR “hepatic echinococcus cyst*”[Title/Abstract] OR “liver echinococcosis”[Title/Abstract] OR “liver hydatid disease”[Title/Abstract]) AND (“Recurrence”[MeSH Terms] OR “recurrence*”[Title/Abstract] OR “relapsing disease”[Title/Abstract] OR “recrudescence*”[Title/Abstract] OR “relapse*”[Title/Abstract] OR “recurrent*”[Title/Abstract] OR “Secondary echinococcosis”[Title/Abstract])) NOT (“case reports”[Publication Type] OR “case report”[All Fields])) AND (humans[Filter])

The search will be updated every six months, or more frequently if necessary. The most recent search was conducted on May 7, 2025.

**Data management and selection process:** Articles retrieved from each source will be managed using COVIDENCE® software, where duplicates will be removed automatically and manually. A pilot test will be conducted with 5–10 articles to evaluate inter-rater agreement on the eligibility criteria.

Independently, two reviewers screened titles and abstracts. Full texts of potentially eligible studies will be retrieved, and two authors will review independently. Each article will require two votes for inclusion.

Discrepancies at any stage will be resolved by consensus between reviewers, and if necessary, a third reviewer will be consulted.

**Data collection process:** Two independent reviewers will perform data extraction using a standardized form based on the CHARMS-PF tool [26] and adapted to the variables of interest. This form will be piloted with 3–5 primary studies. Inconsistencies in the extraction will be resolved by consensus among reviewers.

**Data items:** The extraction form includes the items: article information, participants, sample size, CE recurrence, PFs, effect sizes, statistical analysis, missing data, and conclusions. For more information, view S2 Table.

**Unit of analysis:** Patients undergoing surgery for HCE will be the unit of analysis. Odds ratios (OR) will be the primary measure of association. Reported OR, relative risks (RR), and hazard ratios (HR) from bivariate analysis will be extracted and converted to OR at specific time points [27].

**Treatment of missing data:** We will contact study authors to retrieve missing or unclear data to improve accuracy and reduce the risk of reporting bias. This includes missing bias assessments, intervention details, or outcome data.

When PFs are reported as dichotomous variables, 2x2 tables will be constructed, and converted to OR with 95% CI, applying the Haldane-Anscombe correction in cases with zero cell frequencies [28]. Associations will be recalculated for consistency. We will synthesize independently adjusted (multivariable) and unadjusted (univariable) associations.

We will include studies reporting associations between PFs and recurrence, even if effect sizes are missing or only described as “non-significant.” When needed, we will estimate effect sizes from available data (e.g., 2x2 tables, figures) using indirect estimation methods as described by Parmar (1998) and Tierney (2007) [29,30].

**Outcomes and prioritization:** The initial search will be open-ended and including all variables reported as potential PFs. The primary outcome will be the recurrence of CE, defined as the appearance of new intra-abdominal cysts after curative treatment, at any point during follow-up. In addition, the definitions proposed in the primary articles included were adopted.

**Risk of bias in individual studies:** Two independent reviewers will assess risk of bias using the Quality in Prognosis Studies (QUIPS) tool [31]. This instrument evaluates six domains: study participation, attrition, prognostic factor measurement, outcome measurement, confounding, and statistical analysis/reporting. Each domain will be rated as low, moderate, or high risk of bias, considering prompting items to guide the judgment (S3 Table). Discrepancies will be resolved by consensus.

The overall risk of the study will be classified as low risk, with most domains present low risk, and none are high risk. Moderate risk when two or more areas present moderate risk and none present high risk. And high risk, with one or more domains being at high risk.

**Methodological quality assessment:** Two independent authors will assess methodological quality (MQ) using the MInCir-Pr_2_ tool [32]. This validated instrument includes four domains: study design, population size, methodology, statistical analysis, and conclusions. Scores range from 7 to 60. The average score ≥33 will indicate adequate MQ, while ≤32 will indicate inadequate MQ (Table 4).

**Table 4:** Domains and score of the MInCir-Pr_2_ tool. Adapted by Manterola et. al. [32].

Domains and items		Score
**Domain 1: Study design ** ^Ϯ^		
Prospective cohort study or RCT double-masked		15
Retrospective cohort study or Not RCT, single, or absent masked		10
Case-control study		8
Cross-sectional study		6
Case series or Case report		3
Domain 2: Study population x justification factor* ^Ϯ^		
n > 501		7 or 15*
n = 201–500		6 or 12*
n = 151–200		5 or 10*
n = 101–150		4 or 8*
n = 51–100		3 or 6*
n = 31–50		2 or 4*
n ≤ 30		1 or 2*
**Domain 3: Methodology**		
*Objectives* ^*Ϯ*^	Clear, precise, and concrete objectives	3
	Vague objectives	2
	No objectives	1
*Design used* ^*Ϯ*^	The type of design used is identified	3
	The design is not clear, or not mentioned	1
*Variables* ^*¥*^	Outcome variable adequately defined	1
	The exposure variable is adequately defined	1
	Confounding variables are adequately defined	1
*Sample size* ^*Ϯ*^	Includes an estimate or calculation of sample size	3
	Does not include the estimation or calculation of sample size.	1
*Follow-up* ^*¥*^	Mentions percentage loss to follow-up of cohort(s)	1
	Follow-up of cohort(s) is greater than 80%	1
	Causes for the loss to follow-up of the cohort(s) are explained	1
**Domain 4: Analysis and conclusions** ^***¥***^		
Includes calculation of risk measures		5
Reported data allow calculation of risk measures		2
Includes predictive or association models		5
There is consistency between the objective, the methodology, and the results		3
**Total**		**7–60**

RCT: Randomized clinical trial. ^Ϯ^: Only one election. ^¥^: Evaluate each item independently.

*Justification factor: Studies that estimate the sample size used are double-scored.

**Synthesis methods:** We will perform a qualitative and descriptive synthesis of the evidence. Statistical analysis will use the R software version 4.4.2 and the metafor package.

The primary outcome will be analyzed using random-effects meta-analysis. We will use the Restricted Maximum Likelihood (REML). OR and their precision (e.g., 95% CI, standard error) will be transformed into natural logarithms.

**Heterogeneity assessment:** Given expected clinical and methodological variability, we anticipate substantial heterogeneity. We will assess heterogeneity using Chi², Tau², and the I² statistic, and estimate 95% prediction intervals for each outcome. I² will be interpreted as follows: low or unimportant heterogeneity (0–40%), moderate heterogeneity (30–60%), substantial heterogeneity (50–90%), and considerable heterogeneity (75–100%).

**Reporting bias assessment:** Publication bias will be assessed in outcomes reported in ≥10 studies, we will analyze funnel plot asymmetry and apply Egger’s test. Considering as null hypothesis that there is no publication bias, a p-value ≤0.05 will indicate potential publication bias.

**Certainty assessment:** Two independent reviewers will assess the certainty of evidence using the Grades of Recommendation, Assessment, Development and Evaluation (GRADE) approach adapted for prognostic factor research [33]. Certainty will be graded as high, moderate, low, or very low, relationship in study design and internal validity across five domains: risk of bias, inconsistency, imprecision, indirectness, and publication bias.

## Discussion

Understanding PF in the clinical setting allows health care providers to stratify patients by baseline characteristics, permitting individualized treatment and further follow-up based on their baseline risk [34].

Applying this to the study of predictive factors for the recurrence of HCE, a disease considered a neglected disease by the WHO and a research priority by the Pan American Health Organization (PAHO) [1,35]. It is of great relevance, particularly in countries where CE is endemic. However, due to the amount of information and the heterogeneity available, its interpretation is difficult; therefore, a focused systematic review (SR) is essential to reduce uncertainty.

The findings of this SR will help to identify FPs with recurrence, contributing to a more accurate risk stratification of patients and to a better allocation of healthcare resources. Similarly, the results may support the development of risk prediction tools for use in clinical practice and guide the design and analysis of future clinical trials, especially in a field where methodological quality is often poor, risk of bias is high, and certainty of evidence is low [36,37].

## Supporting information

S1 Table PRISMA-P checklist. (DOCX)

S2 Table Data extraction form. (XLSX)

S3 Table Prompting items QUIPS. PF: Prognostic factor. RoB: Risk of Bias.
(XLSX)
